# Association of hospital volume with conversion to open from minimally invasive colectomy in patients with diverticulitis: A national analysis

**DOI:** 10.1371/journal.pone.0284729

**Published:** 2023-04-28

**Authors:** Shayan Ebrahimian, Arjun Verma, Sara Sakowitz, Manuel Orellana Olmedo, Nikhil Chervu, Aimal Khan, Alexander Hawkins, Peyman Benharash, Hanjoo Lee

**Affiliations:** 1 Division of Cardiac Surgery, Cardiovascular Outcomes Research Laboratories, David Geffen School of Medicine at UCLA, Los Angeles, CA, United States of America; 2 Department of Surgery, Harbor-UCLA Medical Center, Torrance, CA, United States of America; 3 Department of Surgery, Vanderbilt University Medical Center, Nashville, TN, United States of America; Children’s National Hospital, UNITED STATES

## Abstract

**Background:**

Despite the known advantages of minimally invasive surgery (MIS) for diverticular disease, the impact of conversions to open (CtO) colectomy remains understudied. The present study used a nationally representative database to characterize risk factors and outcomes associated with CtO in patients with diverticular disease.

**Methods:**

All elective adult hospitalizations entailing colectomy for diverticulitis were identified in the 2017–2019 Nationwide Readmissions Database. Annual institutional caseloads of MIS and open colectomy were independently tabulated. Restricted cubic splines were utilized to non-linearly estimate the risk-adjusted association between hospital volumes and CtO. Additional regression models were developed to evaluate the association of CtO with outcomes of interest.

**Results:**

Of an estimated 110,281 patients with diverticulitis who met study criteria, 39.3% underwent planned open colectomy, 53.3% completed MIS, and 7.4% had a CtO. Following adjustment, an inverse relationship between hospital MIS volume and risk of CtO was observed. In contrast, increasing hospital open volume was positively associated with greater risk of CtO. On multivariable analysis, CtO was associated with lower odds of mortality (AOR 0.3, p = 0.001) when compared to open approach, and similar risk of mortality when compared to completed MIS (AOR 0.7, p = 0.436).

**Conclusion:**

In the present study, institutional MIS volume exhibited inverse correlation with adjusted rates of CtO, independent of open colectomy volume. CtO was associated with decreased rates of mortality compared to planned open approach but equivalence risk relative to completed MIS. Our findings highlight the importance of MIS experience and suggest that MIS may be safely pursued as the initial surgical approach among diverticulitis patients.

## Introduction

Three decades of experience has shown the safety and efficacy of minimally invasive approaches to a multitude of abdominal operations [1–4]. For diverticular disease, in particular, minimally invasive surgery (MIS) has demonstrated substantial reductions in perioperative complications, mortality, and costs, compared to open colectomy [5–10]. Nonetheless, questions remain regarding the impact of unplanned conversions from elective MIS to open colectomy, which is reported to occur in up to 42% of cases [7, 11, 12]. Several studies have reported conversion to open (CtO) to be independently associated with decreased overall survival among patients undergoing elective colectomy for colon cancer [4, 13, 14]. Yet, others have not noted such a survival decrement [15, 16].

A number of patient factors including male sex, older age, obesity, and smoking, have been associated with increased risk of CtO in colectomy [17, 18]. Additionally, institutional case volume has been shown to be a significant risk factor for CtO across several operations [18–22]. Surgeon experience and streamlined care pathways are hypothesized to contribute to the observed volume-outcome relationship [18, 21]. Such factors are particularly relevant in the setting of diverticulitis where inherent inflammation and associated phlegmon, abscesses, or fistulas can increase operative complexity [23]. The relationship between institutional volume and CtO has not been evaluated at a national level among diverticulitis patients.

We used a nationally representative sample to characterize patient and hospital factors associated with the risk of CtO in colectomy for diverticulitis. We hypothesized that after risk adjustment, higher institutional MIS colectomy caseload would be associated with reduced risk of CtO. We further hypothesized CtO would be associated with increased mortality, perioperative complications, hospitalization costs, and unplanned 30-day readmission.

## Materials and methods

### Data source and study population

This was a retrospective cohort study of the 2017–2019 Nationwide Readmission Database (NRD) [24]. The largest all-payer readmissions database, the NRD is maintained by Healthcare Cost and Utilization Project (HCUP) and provides accurate estimates for ~60% of all US hospitalizations [24]. The NRD contains unique linkage identifiers which allow patients to be tracked across hospitals within a calendar year.

**Fig 1:**
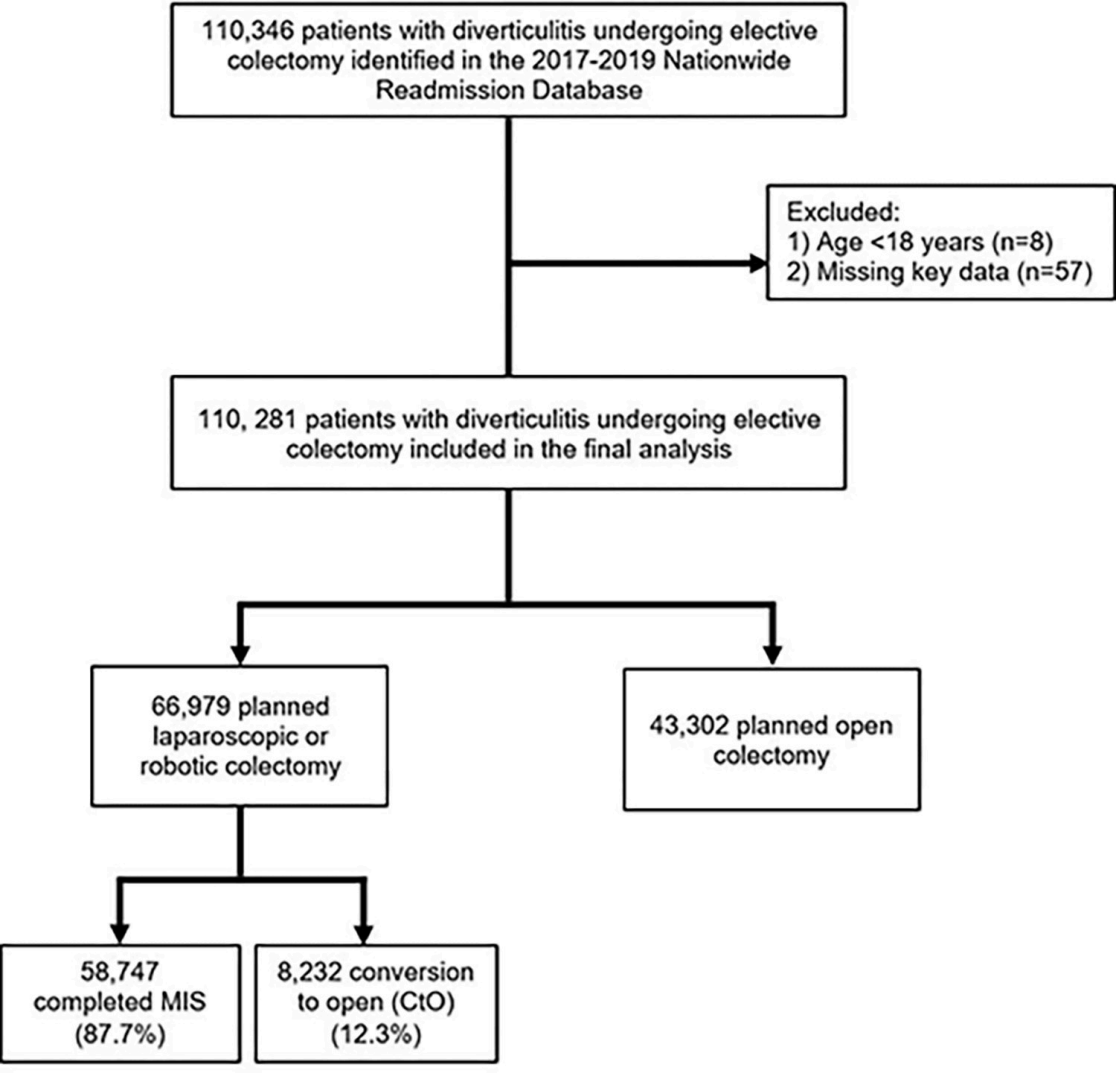
Flow chart of the patient population selection process.

All elective adult (≥18 years) hospitalizations entailing colectomy for diverticulitis were tabulated using previously published International Classification of Diseases, Tenth Revision (ICD-10) diagnosis codes [25–27]. Patients were classified into three cohorts of completed minimally invasive colectomy (*MIS*), laparoscopic or robotic colectomy converted to open (*CtO*), and planned open colectomy (*Open*) using relevant ICD-10 codes. Records missing key information (age, sex, in-hospital mortality, and hospitalization costs) were excluded (<1%; Fig 1).

### Variable definitions and study outcomes

Demographic and clinical characteristics were defined according to the NRD data dictionary and were compared between the three cohorts. Demographic characteristics included age, sex, income quartile, and insurance payer. Clinical characteristics included the van Walraven modification of the Elixhauser Comorbidity Index, a validated composite of 30 comorbidities that provides a numeric estimate of chronic conditions [28]. Other comorbidities, such as congestive heart failure, history of tobacco use, obesity, and cancer, as well as the anatomic extent of resection were tabulated [29]. Perioperative adverse events, such as cardiac, gastrointestinal, and infectious complications as well as ileostomy and colostomy formation were ascertained using ICD-10 codes [29]. Intraoperative accidental puncture of organ and intraoperative hemorrhage were also ascertained. Acute venous thromboembolism (VTE) was tabulated using codes for acute pulmonary embolism and deep venous thrombosis [30]. Hospitalization costs were calculated utilizing the hospital specific cost-to-charge ratios provided by HCUP and inflation adjusted to 2019 Personal Health Care Price Index [24].

Institutional MIS volume was calculated as the laparoscopic and robotic colectomy caseload during each calendar year. Hospital open colectomy caseload (open volume) was derived from the number of open colectomy performed each year, while CtO cases were not considered in either the MIS or open volume calculations. MIS and open volume estimates were computed independently for each year as the NRD does not track hospitals across calendar years.

The primary endpoints of this study were risk-adjusted rates of CtO based on annual hospital MIS and open colectomy volume. The secondary endpoints were hospital mortality, perioperative complications, length of stay (LOS), hospitalization costs, non-home discharge, and 30-day unplanned readmissions.

### Statistical analysis

Categorical variables are reported as proportion and compared using the Pearson’s Χ^2^ test. Continuous variables with approximately normal distribution are summarized as means with standard deviation (SD) and compared using the Adjusted Wald test. Continuous variables with skewed distributions are reported as medians with interquartile range (IQR) and compared using Mann-Whitney U test. A multivariable logistic regression was developed to assess the association of patient and operative factors with CtO. Hospital colectomy volume was modeled as a restricted cubic spline to allow for the presence of nonlinearity. Variable selection regression models were guided by elastic net regularization, an automated method that reduced collinearity and increases out-of-sample validity [31]. The area under the receiver-operating characteristic curve (C-statistic) were utilized to evaluate final models. Variance inflation factor (VIF) was utilized to assess collinearity between annual MIS and open volume. A VIF less than 10 was yielded, and thus MIS and open hospital volume were retained as covariates in all regressions [32]. To account for potential intergroup differences, we used entropy balancing as sensitivity analysis. Entropy balancing retains the entire cohort for analysis while addressing covariate balance between groups [33]. Additionally, hospitals were classified into low-, medium- and high-volume tertiles (LVH, MVH and HVH, respectively) and a sensitivity analysis of our risk-adjusted results was performed within LVH and HVH. Regression outputs are reported as beta coefficients (β) or adjusted odds ratios (AOR) with 95% confidence intervals (95% CI). Statistical significance was defined as p<0.05, and all analyses were performed using Stata software version 16.0 (StataCorp LP, College Station, TX). This study was deemed exempt from full review by the Institutional Review Board at the University of California, Los Angeles (IRB# 17–001112). Patient consent was also waived due to the de-identified nature of the NRD.

## Results

### Demographic comparison

Of an estimated 110,281 patients with diverticulitis who met study criteria, 39.3% underwent planned open colectomy (*Open*), 53.3% completed laparoscopic or robotic colectomy (*MIS*), and 7.4% had a CtO. The conversion rate among all intended minimally invasive colectomy was 12.3%. Compared to *MIS* and *Open*, those in the *CtO* cohort were older, more commonly insured by Medicaid, and had a higher prevalence of hypertension, obesity, and tobacco use (Table 1). Compared to others, *MIS* patients had the lowest estimated burden of comorbidities as measured by the Elixhauser Index (Table 1).

**Table 1:** Comparison of baseline patient characteristics of patients with diverticulitis undergoing elective colectomy, stratified by operative approach.

Parameter	MIS	CtO	Open	P value
	n = 58,747	n = 8,232	n = 43,302	
Age, median [IQR], year	58 [50–67]	62 [53–70]	61 [52–70]	<0.001
Female, n (%)	33,172 (56.5)	4,699 (57.1)	24,925 (57.6)	0.053
Income level percentile, n (%)				<0.001
76^th^-100^th^	16,508 (28.1)	1,877 (22.8)	9,267 (21.4)	
51^st^-75^th^	17,154 (29.2)	2,297 (27.9)	11,302 (26.1)	
26^th^-50^th^	14,804 (25.2)	2,511 (30.5)	12,428 (28.7)	
0-25^th^	10,281 (17.5)	1,539 (18.7)	10,306 (23.8)	
Insurance type, n (%)				<0.001
Private	34,073 (58.0)	3,893 (47.3)	20,742 (47.9)	
Medicare	19,092 (32.5)	3,440 (41.8)	17,884 (41.3)	
Medicaid	3,525 (6.0)	600 (7.3)	2,988 (6.9)	
Other Payer	2,056 (3.5)	304 (3.7)	1,646 (3.8)	
Location of resection, n (%)				<0.001
Right hemicolectomy	484 (0.8)	79 (0.9)	443 (1.0)	
Transverse colectomy	168 (0.3)	23 (0.2)	213 (4.9)	
Left hemicolectomy	2,685 (4.6)	565 (6.9)	3,025 (7.0)	
Sigmoidectomy	54,893 (93.4)	7,458 (90.6)	38,943 (89.9)	
Total/subtotal colectomy	517 (0.9)	106 (1.3)	679 (1.6)	
Intraoperative hemorrhage, n (%)	42 (0.1)	41 (0.5)	54 (0.1)	<0.001
Intraoperative accidental puncture, n (%)	392 (0.7)	270 (3.3)	566 (1.3)	<0.001
Elixhauser Comorbidity Index, median [IQR]	1 [0–2]	2 [1–3]	2 [1–3]	<0.001
Comorbidities, n (%)				
Congestive heart failure	1,345 (2.3)	320 (3.9)	1,824 (4.2)	<0.001
Coronary artery disease	4,214 (7.2)	678 (8.2)	4,105 (9.5)	0.01
Hypertension	26,711 (45.5)	4,328 (52.6)	22,379 (51.7)	<0.001
Tobacco use	7,706 (13.1)	1,301 (15.8)	6,410 (14.8)	<0.001
Pulmonary circulatory disorders	296 (0.5)	93 (1.1)	510 (1.2)	<0.001
Chronic pulmonary disease	8,774 (14.9)	1,430 (17.4)	7,466 (17.2)	<0.001
Peripheral vascular disorder	1,399 (2.4)	278 (3.4)	1,487 (3.4)	<0.001
Diabetes	7,081 (12.0)	1,201 (14.6)	6,376 (14.7)	<0.001
Liver disease	1,418 (2.4)	244 (3.0)	1,145 (2.6)	0.06
Coagulopathy	851 (1.4)	143 (1.7)	919 (2.1)	0.17
Anemia	758 (1.3)	145 (1.8)	959 (2.2)	0.017
Cancer	1,087 (1.8)	266 (3.2)	1,620 (3.7)	<0.001
Obesity	11,905 (20.3)	1,972 (23.9)	8,965 (20.7)	<0.001
Fluid and electrolyte disorders	5,051 (8.6)	1,346 (16.3)	6,702 (15.5)	<0.001
Renal failure	2,168 (3.7)	485 (5.9)	2,567 (5.9)	<0.001

MIS: completed minimally invasive colectomy; CtO: minimally invasive colectomy converted to open; Open: planned open colectomy; SD: Standard Deviation; IQR: interquartile range.

### Association of patient factors and hospital volume with CtO

**Fig 2:**
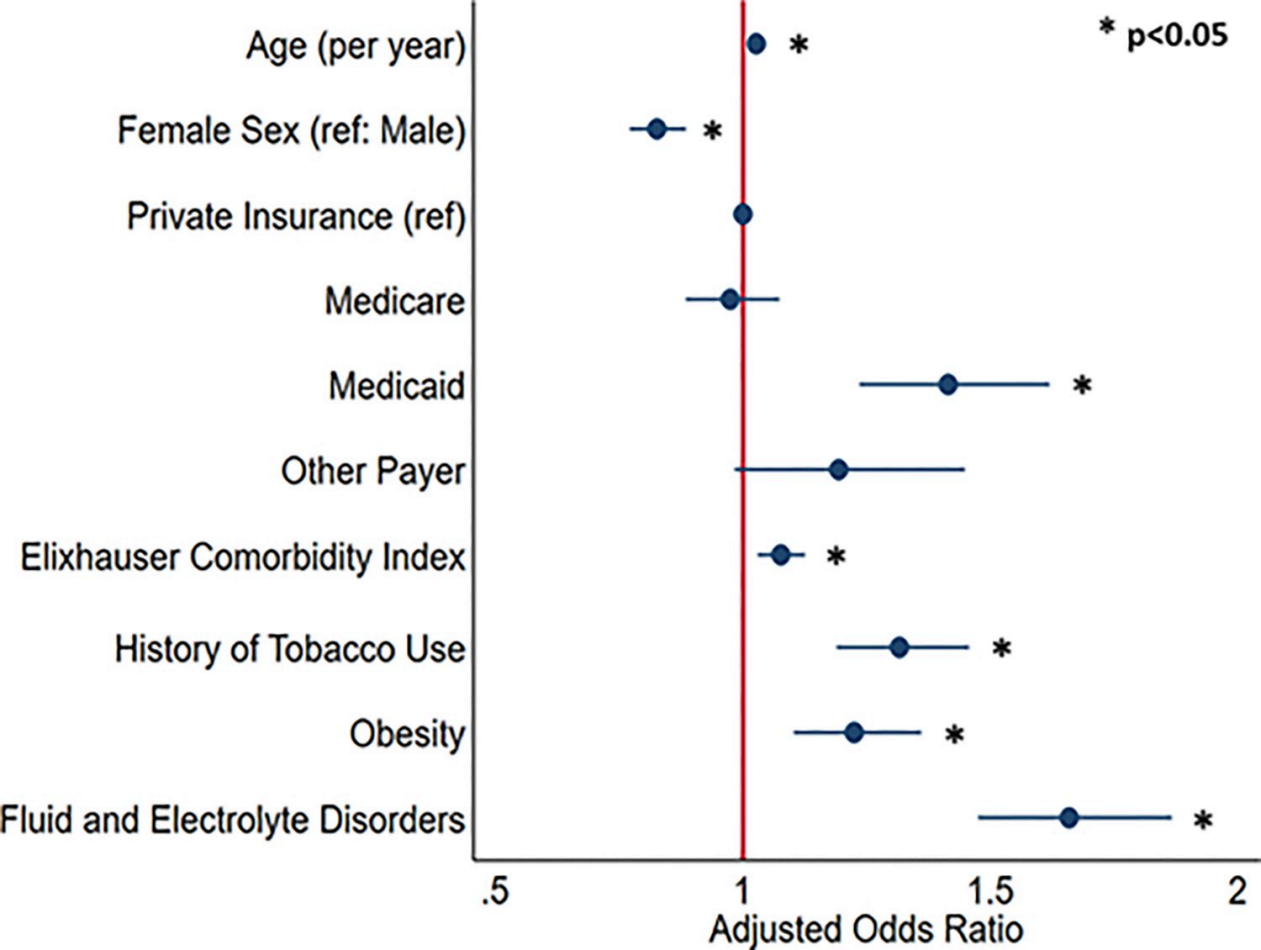
Patient characteristics associated with risk of conversion to open following intended minimally invasive colectomy.

A multivariable logistic regression model (C-statistic: 0.67) was developed to predict patient factors associated with CtO. After adjustment for intergroup differences, advancing age (AOR 1.03/year, 95%CI 1.02, 1.03), Medicaid insurance (AOR 1.41, 95%CI 1.23, 1.61), and increasing in Elixhauser index (AOR/point 1.08, 95%CI 1.03, 1.13) were associated with greater likelihood of CtO. Additionally, patients with obesity (AOR 1.22, 95%CI 1.10, 1.36), fluid and electrolyte disorders (AOR 1.62, 95%CI 1.44, 1.82), and history of tobacco use (AOR 1.31, 95%CI 1.19, 1.45) had greater odds of CtO (Fig 2). Intraoperative accidental puncture (AOR 4.63, 95% CI 3.64, 5.89) and hemorrhage (AOR 6.70, 95% CI 3.70, 12.15) were also significantly associated with increase in likelihood of conversion.

**Fig 3:**
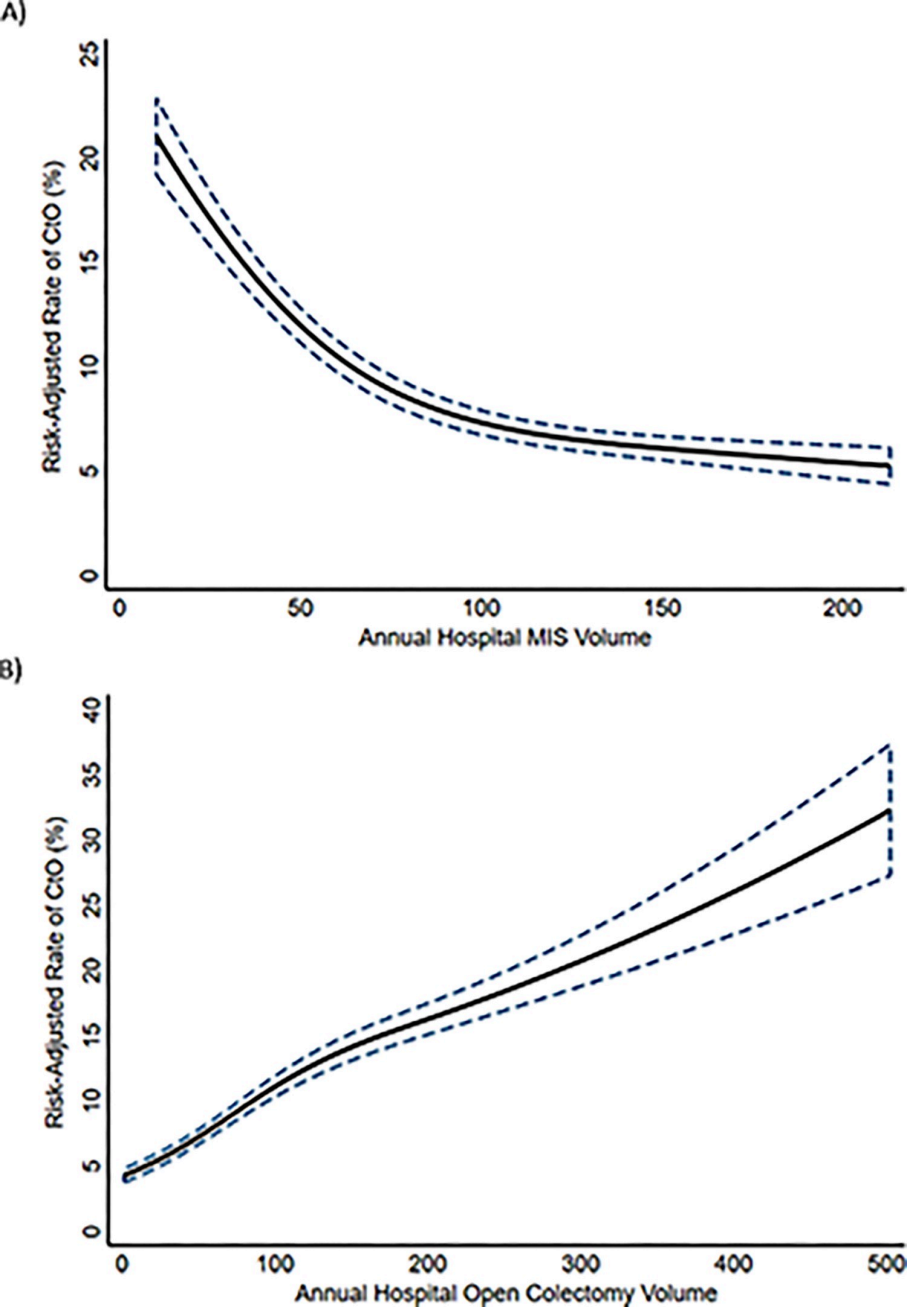
Relationship between annual hospital colectomy volume modeled using cubic splines (A: minimally invasive surgery (MIS); B: Open) and risk-adjusted rates of conversion to open (CtO).

**Fig 4:**
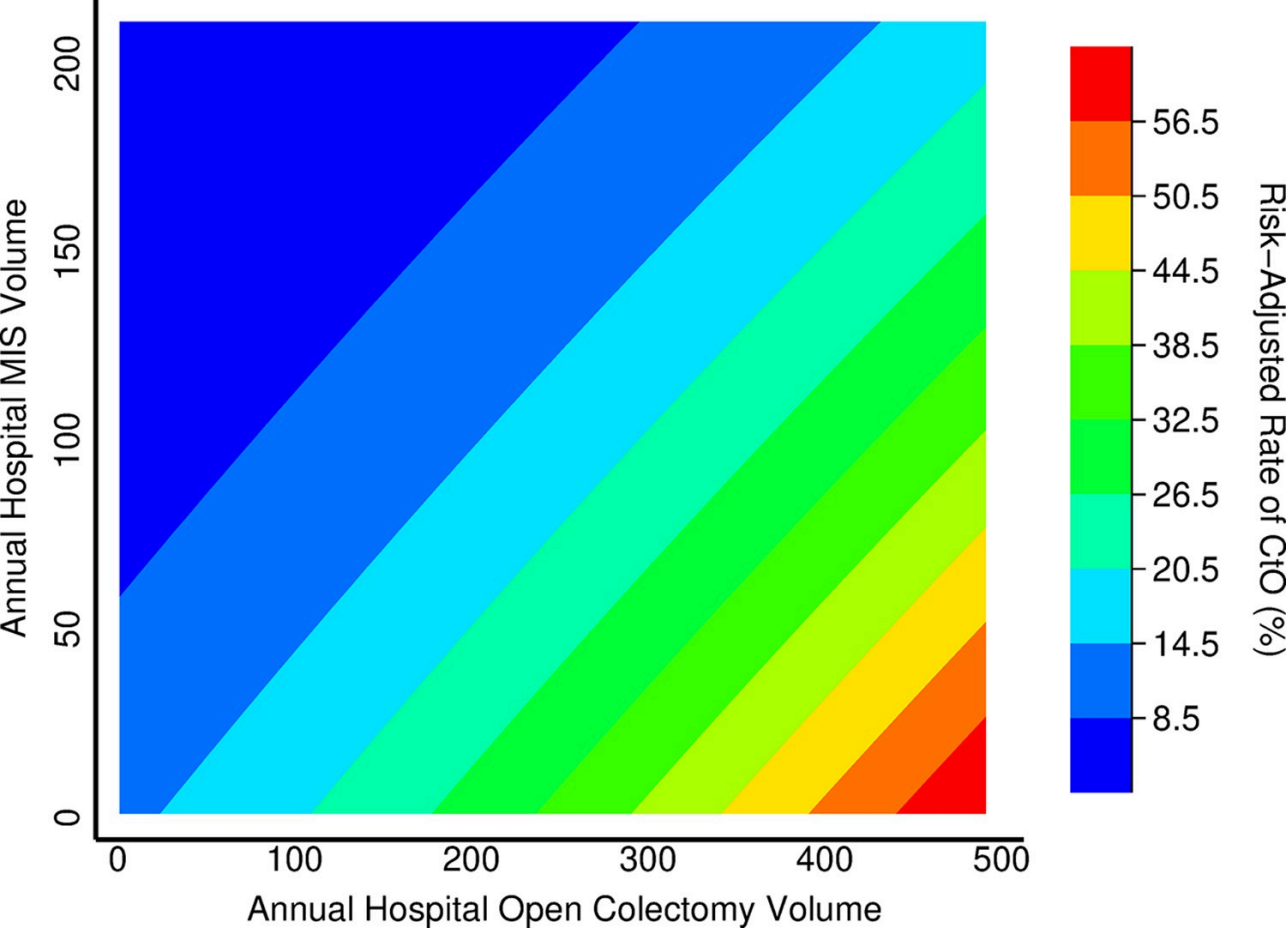
Contour plot of relationship between conversion to open (CtO) and annual hospital volume of minimally invasive surgery (MIS) and open colectomy.

The association of hospital volume and the risk of CtO was analyzed using a continuous approach. Following adjustment, restricted cubic spline estimation demonstrated that a significant and inverse relationship between hospital MIS volume and risk of CtO (Fig 3A). In contrast, increasing hospital open volume was positively associated with greater risk of CtO (Fig 3B). Fig 4 demonstrates that independent of institutional open volume, increasing hospital MIS volume is associated with reduced rates of CtO.

### Unadjusted outcomes following colectomy

Compared to *MIS* and *CtO* patients, the *Open* cohort demonstrated the greatest rates of in-hospital mortality (*MIS*: 0.1%, *CtO*: 0.2%, *Open*: 0.6%, p<0.001) and acute VTE as well as cardiac and respiratory complications (Table 2). In contrast, those requiring CtO exhibited the highest rates of ileostomy formation as well as gastrointestinal and infectious complications compared to the *MIS* and *Open* cohorts (Table 2). Compared to others, the *CtO* cohort accumulated the greatest hospitalization costs and had increased rates of 30-day, unplanned readmissions (Table 2).

**Table 2:** Crude outcomes for patients with diverticulitis undergoing elective colectomy stratified by operative approach.

	MIS	CtO	Open	P Value
	n = 58,747	n = 8,232	n = 43,302	
Clinical Outcomes (%)				
In-Hospital Mortality	0.1	0.2	0.6	<0.001
Cardiac Complications	0.3	0.5	0.7	<0.001
Acute VTE	0.2	0.8	0.9	<0.001
Respiratory Complications	1.3	3.5	3.9	<0.001
Gastrointestinal Complications	0.3	1.1	0.7	<0.001
Infectious Complications	2.2	4.8	4.5	<0.001
Ileostomy formation	3.3	12.5	6.4	<0.001
Colostomy formation	1.2	4.7	4.8	0.006
Resource Utilization (median, IQR)				
Length of Stay (days)	3 [2–4]	5 [4–7]	5 [3–7]	<0.001
Hospitalization Costs ($1,000s)	16.6 [12.8–22.3]	20.6 [15.4–29.3]	17.0 [12.8–23.5]	<0.001
Non-home Discharge (%)	1.8	5.3	6.2	<0.001
30-day, Unplanned Readmissions (%)	5.7	9.0	7.3	<0.001

MIS: completed minimally invasive colectomy; CtO: minimally invasive colectomy converted to open; Open: planned open colectomy; IQR: interquartile range.

### Adjusted outcomes of CtO compared to MIS

With *MIS* as reference, the *CtO* cohort did not yield a statistically different risk of in-hospital mortality (AOR 0.7, 95%CI 0.3, 1.7, p = 0.436). However, the *CtO* cohort exhibited increased likelihood of acute VTE, ostomy formation as well as respiratory, gastrointestinal, and infectious complications, compared to *MIS* patients (Table 3). Notably, CtO was associated with 1.8-day increment in LOS and +$4,200 in hospitalization costs. The *CtO* cohort also demonstrated increased odds of non-home discharge and 30-day, unplanned readmission when compared to *MIS* patients (Table 3).

**Table 3:** Risk-adjusted outcomes for patients with diverticulitis undergoing elective colectomy stratified by operative approach.

	MIS	CtO	^a^P value	Open	CtO	^b^P value
Clinical Outcomes, AOR (95% CI)						
In-Hospital Mortality	Ref	0.7 [0.3, 1.7]	0.4	Ref	0.3 [0.1, 0.6]	0.001
Cardiac Complications	Ref	1.0 [0.6, 1.8]	0.9	Ref	0.7 [0.5, 1.1]	0.2
Acute VTE	Ref	1.9 [1.1, 3.3]	0.02	Ref	0.8 [0.5, 1.3]	0.4
Respiratory Complications	Ref	1.8 [1.4, 2.2]	<0.001	Ref	0.9 [0.8, 1.1]	0.5
Gastrointestinal Complications	Ref	2.7 [1.8, 4.0]	<0.001	Ref	1.4 [1.0, 2.0]	0.04
Infectious Complications	Ref	1.8 [1.5, 2.2]	<0.001	Ref	1.1 [0.9, 1.2]	0.5
Ileostomy Formation	Ref	3.7 [3.2, 4.2]	<0.001	Ref	2.0 [1.8, 2.3]	<0.001
Colostomy Formation	Ref	3.2 [2.6, 4.0]	<0.001	Ref	1.0 [0.8, 1.2]	0.9
Resource Utilization, AOR/ ß-Coef [95%CI]						
Length of Stay (days)	Ref	1.8 [1.7, 2.0]	<0.001	Ref	0.3 [0.2, 0.5]	<0.001
Hospitalization Costs ($1,000s)	Ref	4.2 [3.7, 4.7]	<0.001	Ref	3.0 [2.4, 3.6]	<0.001
Non-home Discharge	Ref	1.9 [1.6, 2.3]	<0.001	Ref	0.8 [0.7, 1.0]	0.06
30-day, Unplanned Readmissions	Ref	1.4 [1.3, 1.6]	<0.001	Ref	1.2 [1.1, 1.4]	<0.001

MIS: completed minimally invasive colectomy; CtO: minimally invasive colectomy converted to open; Open: planned open colectomy. Risk-adjusted estimates are reported as adjusted odds ratio (AOR) or ß-coefficients with 95% confidence intervals (CI) for binary and continuous variables, respectively.

^a^CtO vs. MIS

^b^CtO vs. Open

### Adjusted outcomes of CtO compared to planned open colectomy

The *CtO* cohort was associated with lower risk of in-hospital mortality (AOR 0.3, 95%CI 0.1, 0.6, p = 0.001) when compared to patients who underwent planned open surgery. The *CtO* cohort exhibited similar odds of acute VTE, colostomy formation, as well as respiratory and cardiac complications (Table 3). However, CtO demonstrated a significant, positive association with the occurrence of gastrointestinal complications and ileostomy formation when compared to *Open* patients (Table 3). The *CtO* cohort was also associated with 0.3-day increment in LOS and +$3,000 in hospitalization costs as well as greater odds of 30-day, unplanned readmission (Table 3).

### Sensitivity analysis

Standard mean differences reporting covariate balance before and after entropy balancing are shown in S1 and S2 Figs. Sensitivity analysis after entropy balancing exhibited similar outcomes of CtO (S1 Table). Sensitivity analysis within LVH and HVH also exhibited similar conclusions as original analysis (S2 and S3 Tables).

### Discussion

Despite technical advances of MIS in the past three decades, CtO remains a significant concern. In line with prior studies, nearly 12.3% of patients who underwent intended laparoscopic or robotic colectomy in our nationally representative sample required CtO [34–36]. Upon multivariable adjustment, advanced age, obesity, tobacco use, and electrolyte disorders were associated with increased odds of CtO. Notably, increasing hospital MIS volume was associated with reduced risk-adjusted rates of CtO, independent of institutional open volume. The *CtO* cohort demonstrated similar likelihood of in-hospital mortality when compared to *MIS*. Interestingly, patients who required CtO faced lower odds of in-hospital mortality when compared to patients who underwent intended open surgery. Several of our findings merit further discussion.

There exists significant debate regarding the outcomes of patients who require CtO compared to those who receive open colectomy [37]. In congruence with a prior national inpatient report, our study of patients with diverticulitis found those who required conversion to face lower odds of in-hospital mortality compared to those who underwent intended open colectomy [35]. In contrast, a prospective study of ~500 colon resections for malignancy at two teaching hospitals demonstrated equivalent outcomes between *Open* and *CtO* patients [17]. This variation in literature may, in part, be attributable to differences in scope of initial indication for surgery and limited sample size. In addition, the present study and the report by Moghadamyeghaneh and colleagues [35] include nationwide centers with lower MIS experience, where surgeons may maintain a lower threshold for CtO when challenged by unplanned complications. In line with prior studies, we also observed that CtO did not alter the odds of in-hospital mortality when compared to the *MIS* patients [15, 16]. This finding suggests that MIS may be safely pursued among patients with diverticulitis as the benefits of MIS may outweigh the clinical burden of CtO. Future studies should examine the clinical implication of broadening patient candidacy criteria for MIS.

In congruence with several prior reports, we found CtO to be associated with increased likelihood of gastrointestinal complications, acute VTE, unplanned readmissions, as well as increment in hospitalization costs [14, 34–36]. The increased complications after conversion were most likely due to increased intraoperative blood loss, longer operative times, as well as the more invasive nature of an open surgery [17, 38]. Our findings also highlight the clinical and financial burden that CtO presents in the setting of colectomy for diverticulitis. As such, the identification of risk factors of CtO and development of targeted mitigation strategies is imperative.

In line with goals of reducing the incidence of CtO, several prior studies have reported risk factors for conversion. Similar to our findings, other investigators have shown male sex, obesity (body mass index of above 30), and smoking status to be associated with increased risk of CtO [36, 39]. Male sex is a well-known risk factor for CtO in pelvic surgery [35]. The narrower male pelvis can significantly increase the complexity of the surgery due to fulcrum effect on the laparoscopic instruments [35]. Similarly, obesity has been highlighted as a risk factor for CtO given the associated technical difficulties with dissection and visualization [38]. Although the mechanism through which smoking status confers increased risk of CtO is unclear, surgeons may maintain lower threshold for CtO in smokers due to awareness of the increased risk of perioperative morbidity in these patients. Providers should remain cognizant of abovementioned factors and counsel patients accordingly in the preoperative period, such as encouraging smoking cessation and weight loss.

Patient factors aside, the present study also identified institutional MIS volume to be associated with significantly reduced risk-adjusted rates of CtO. We demonstrated that even at centers with significant open colectomy volume, low MIS volume is associated with greater likelihood of risk-adjusted CtO. Although not directly measured in the present study, this finding is most likely attributable to surgeon MIS expertise. Prior investigations have demonstrated CtO as an effective proxy for surgical experience, with some defining the end of the learning curve at 55 laparoscopic right-sided colonic resections and 62 cases for left hemicolectomy [17, 18, 22, 40]. However, it is also possible that institutional protocols play a role in reducing rates of CtO at centers with high MIS volume. In particular, these centers may have more refined patient selection criteria to exclude patients at high risk for MIS. Thorough preoperative evaluation for risks of establishing pneumoperitoneum particularly in patients with diminished cardiopulmonary function, hemodynamic instability, or obesity may also optimize MIS outcomes. Nonetheless, our findings demonstrate the need for identification and dissemination of best practices at high MIS volume centers in a concerted effort to reduce the incidence of CtO on a national scale.

The current study has several important limitations inherent to its retrospective design and the use of an administrative database. Causal inferences cannot be drawn due to the observational nature of our study design. Although the NRD is the largest all-payer readmission database, it is susceptible to coding errors and lack granular data such as preoperative laboratory values, imaging, the extent of resection, and operative complexity. We were unable to stratify our analysis by laparoscopy and robotic colectomy. We were also unable to determine surgeon’s experience and preference of initial operative approach as well as the primary indication for CtO. The NRD database also does not contain data regarding history of multiple abdominal surgery and intra-abdominal adhesive disease which may have influenced complexity of the surgical operation and surgeon’s preference of initial operative approach and decision to convert. We could not examine variation in hospital/surgeon expertise over time as the NRD does not track either hospitals or surgeons across years. The increased rates of ileostomy formation observed in the *CtO* cohort may potentially reflect the operator’s concern with regard to the integrity of anastomosis in the setting of the unplanned CtO. However, we were unable to ascertain the presence of anastomosis and evaluate the complications associated with creation and closure of ostomy. Despite these limitations, we utilized robust statistical methods and adhered to appropriate data practices as recommended by HCUP to report nationally representative outcomes.

## Conclusion

The present study used a national cohort of colectomy for diverticulitis to comprehensively evaluate the incidence of, risk factors for and outcomes following CtO. Patient risk factors for CtO included male sex, obesity, fluid and electrolyte disorders and history of tobacco use. Notably, institutional MIS volume exhibited a strong, inverse correlation with risk-adjusted rates of CtO, independent of open colectomy volume. Our findings suggest that MIS may be safely pursued among diverticulitis patients as the benefits of MIS may outweigh risks of unplanned CtO. Nonetheless, given that CtO was associated with significant gastrointestinal complications and financial burden, national efforts to improve minimally invasive training may reduce conversion rates.

## Supporting information

S1 Fig Pre- and post-covariate balance among MIS and CTO cohorts after entropy balancing. (TIF)

S2 Fig Pre- and post-covariate balance among CTO and open cohorts after entropy balancing. (TIF)

S1 Table Sensitivity analysis after entropy balancing. Risk-adjusted outcomes for patients with diverticulitis undergoing elective colectomy stratified by operative approach. *MIS*: *completed minimally invasive colectomy; CtO*: *minimally invasive colectomy converted to open; Open*: *planned open colectomy*. *Risk-adjusted estimates are reported as adjusted odds ratio (AOR) or ß-coefficients* with 95% confidence intervals (CI) *for binary and continuous variables*, *respectively*.
(DOCX)

S2 Table Sensitivity analysis: Risk-adjusted outcomes for patients with diverticulitis undergoing elective colectomy at high volume hospitals stratified by operative approach. *MIS*: *completed minimally invasive colectomy; CtO*: *minimally invasive colectomy converted to open; Open*: *planned open colectomy*. *Risk-adjusted estimates are reported as adjusted odds ratio (AOR) or ß-coefficients* with 95% confidence intervals (CI) *for binary and continuous variables*, *respectively*.
(DOCX)

S3 Table Sensitivity analysis: Risk-adjusted outcomes for patients with diverticulitis undergoing colectomy at low volume hospitals stratified by operative approach. *MIS*: *completed minimally invasive colectomy; CtO*: *minimally invasive colectomy converted to open; Open*: *planned open colectomy*. *Risk-adjusted estimates are reported as adjusted odds ratio (AOR) or ß-coefficients* with 95% confidence intervals (CI) *for binary and continuous variables*, *respectively*.
(DOCX)
