# Supplementary figures and images for: Association of hospital volume with conversion to open from minimally invasive colectomy in patients with diverticulitis: A national analysis
Source: PLoS One. 2023 Apr 28;18(4):e0284729. doi: 10.1371/journal.pone.0284729 (PMC10146460; doi:10.1371/journal.pone.0284729)

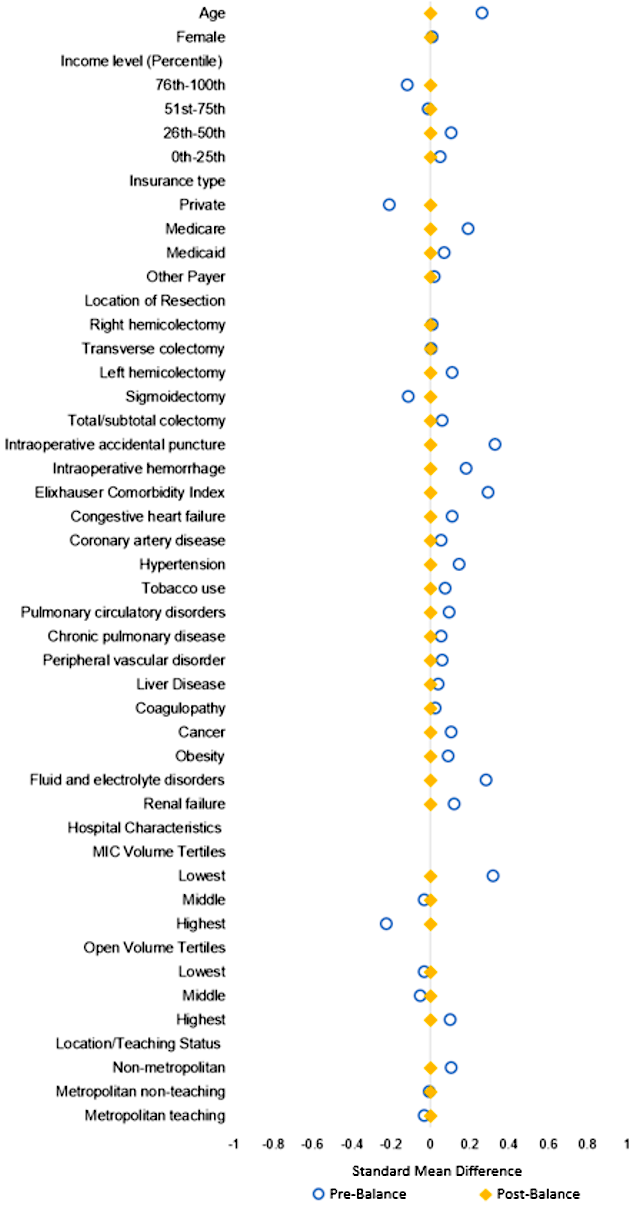

Supplement: S1 Fig — (TIF) [file pone.0284729.s001.tif]

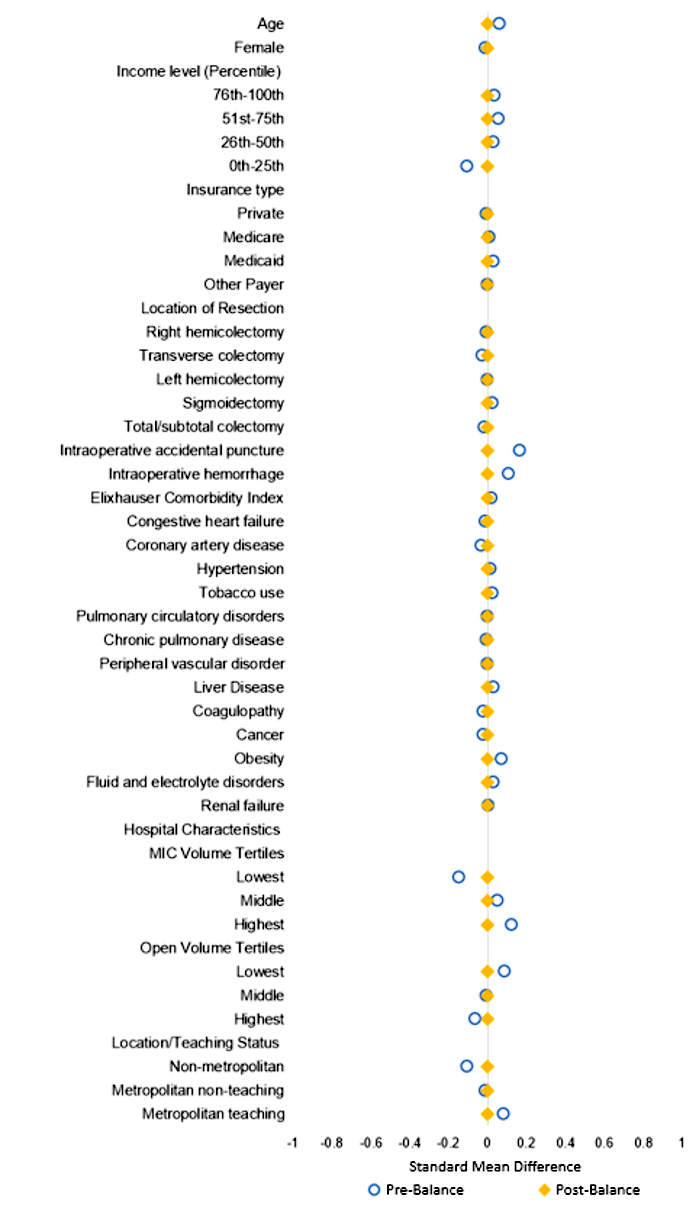

Supplement: S2 Fig — (TIF) [file pone.0284729.s002.tif]
