# Supplementary material for: Association of hospital volume with conversion to open from minimally invasive colectomy in patients with diverticulitis: A national analysis
Source: PLoS One. 2023 Apr 28;18(4):e0284729. doi: 10.1371/journal.pone.0284729 (PMC10146460; doi:10.1371/journal.pone.0284729)
Supplement: S1 Table — Risk-adjusted outcomes for patients with diverticulitis undergoing elective colectomy stratified by operative approach. MIS: completed minimally invasive colectomy; CtO: minimally invasive colectomy converted to open; Open: planned open colectomy. Risk-adjusted estimates are reported as adjusted odds ratio (AOR) or ß-coefficients with 95% confidence intervals (CI) for binary and continuous variables, respectively. (DOCX) [file pone.0284729.s003.docx]

**Supplementary Table S1. Sensitivity Analysis after Entropy Balancing. Risk-adjusted Outcomes for Patients with Diverticulitis Undergoing Elective Colectomy Stratified by Operative Approach.** *MIS: completed minimally invasive colectomy; CtO: minimally invasive colectomy converted to open; Open: planned open colectomy. Risk-adjusted estimates are reported as adjusted odds ratio (AOR) or ß-coefficients* with 95% confidence intervals (CI) *for binary and continuous variables, respectively.*

|  | **MIS** | **CtO** | **^a^P value** | **Open** | **CtO** | **^b^P value** |
| --- | --- | --- | --- | --- | --- | --- |
| Clinical Outcomes, AOR (95% CI) |  |  |  |  |  |  |
| In-Hospital Mortality | Ref | 0.6 [0.2, 1.4] | 0.233 | Ref | 0.2 [0.1, 0.5] | <0.001 |
| Cardiac Complications | Ref | 1.1 [0.6, 1.8] | 0.760 | Ref | 0.7 [0.5, 1.2] | 0.463 |
| Acute VTE | Ref | 1.8 [1.1, 3.1] | 0.021 | Ref | 0.8 [0.5, 1.3] | 0.421 |
| Respiratory Complications | Ref | 1.7 [1.3, 2.1] | <0.001 | Ref | 0.9 [0.7, 1.1] | 0.399 |
| Gastrointestinal Complications | Ref | 2.7 [1.8, 4.0] | <0.001 | Ref | 1.4 [1.0, 2.0] | 0.056 |
| Infectious Complications | Ref | 1.7 [1.4, 2.1] | <0.001 | Ref | 1.0 [0.9, 1.2] | 0.510 |
| Ileostomy Formation | Ref | 3.5 [3.0, 3.9] | <0.001 | Ref | 2.0 [1.8, 2.2] | <0.001 |
| Colostomy Formation | Ref | 2.8 [2.3, 3.5] | <0.001 | Ref | 1.0 [0.8, 1.2] | 0.826 |
| Resource Utilization, AOR/ ß-Coef [95%CI] |  |  |  |  |  |  |
| Length of Stay (days) | Ref | 1.8 [1.6, 1.9] | <0.001 | Ref | 0.3 [0.2, 0.5] | <0.001 |
| Hospitalization Costs ($1,000s) | Ref | 4.1 [3.5, 4.6] | <0.001 | Ref | 2.8 [2.2, 3.5] | <0.001 |
| Non-home Discharge | Ref | 1.7 [1.4, 2.1] | <0.001 | Ref | 0.8 [0.7, 1.0] | 0.041 |
| 30-day, Unplanned Readmissions | Ref | 1.4 [1.2, 1.6] | <0.001 | Ref | 1.2 [1.1, 1.4] | <0.001 |

^a^CtO vs. MIS

^b^CtO vs. Open
