# Supplementary material for: Association of hospital volume with conversion to open from minimally invasive colectomy in patients with diverticulitis: A national analysis
Source: PLoS One. 2023 Apr 28;18(4):e0284729. doi: 10.1371/journal.pone.0284729 (PMC10146460; doi:10.1371/journal.pone.0284729)
Supplement: S2 Table — MIS: completed minimally invasive colectomy; CtO: minimally invasive colectomy converted to open; Open: planned open colectomy. Risk-adjusted estimates are reported as adjusted odds ratio (AOR) or ß-coefficients with 95% confidence intervals (CI) for binary and continuous variables, respectively. (DOCX) [file pone.0284729.s004.docx]

**Supplementary Table S2. Sensitivity Analysis: Risk-adjusted outcomes for patients with diverticulitis undergoing elective colectomy at High Volume Hospitals stratified by operative approach.** *MIS: completed minimally invasive colectomy; CtO: minimally invasive colectomy converted to open; Open: planned open colectomy. Risk-adjusted estimates are reported as adjusted odds ratio (AOR) or ß-coefficients* with 95% confidence intervals (CI) *for binary and continuous variables, respectively.*

|  | **MIS** | **CtO** | **^a^P value** | **Open** | **CtO** | **^b^P value** |
| --- | --- | --- | --- | --- | --- | --- |
| Clinical Outcomes, AOR (95% CI) |  |  |  |  |  |  |
| In-Hospital Mortality | Ref | 0.4 [0.1, 3.5] | 0.4 | Ref | 0.1 [0.02, 0.8] | 0.03 |
| Cardiac Complications | Ref | 1.2 [0.4, 3.6] | 0.7 | Ref | 0.9 [0.4, 2.0] | 0.7 |
| Acute VTE | Ref | 4.6 [2.0, 10] | <0.001 | Ref | 1.4 [0.7, 2.7] | 0.4 |
| Respiratory Complications | Ref | 2.2 [1.5, 3.3] | <0.001 | Ref | 1.2 [0.8, 1.7] | 0.4 |
| Gastrointestinal Complications | Ref | 2.6 [1.3, 5.5] | 0.01 | Ref | 1.6 [0.7, 3.5] | 0.2 |
| Infectious Complications | Ref | 1.9 [1.4, 2.6] | <0.001 | Ref | 1.4 [0.9, 1.9] | 0.06 |
| Ileostomy Formation | Ref | 3.4 [2.7, 4.2] | <0.001 | Ref | 2.1 [1.7, 2.5] | <0.001 |
| Colostomy Formation | Ref | 2.2 [1.4, 3.6] | 0.002 | Ref | 0.9 [0.6, 1.4] | 0.7 |
| Resource Utilization, AOR/ ß-Coef [95%CI] |  |  |  |  |  |  |
| Length of Stay (days) | Ref | 2.0 [1.7, 2.3] | <0.001 | Ref | 0.5 [0.2, 0.9] | 0.005 |
| Hospitalization Costs ($1,000s) | Ref | 4.6 [3.6, 5.7] | <0.001 | Ref | 3.0 [1.8, 4.2] | <0.001 |
| Non-home Discharge | Ref | 2.8 [2.0, 4.1] | <0.001 | Ref | 1.2 [0.9, 1.6] | 0.3 |
| 30-day, Unplanned Readmissions | Ref | 1.6 [1.3, 2.0] | <0.001 | Ref | 1.4 [1.1, 1.7] | 0.002 |

^a^CtO vs. MIS

^b^CtO vs. Open
