# Supplementary material for: Association of hospital volume with conversion to open from minimally invasive colectomy in patients with diverticulitis: A national analysis
Source: PLoS One. 2023 Apr 28;18(4):e0284729. doi: 10.1371/journal.pone.0284729 (PMC10146460; doi:10.1371/journal.pone.0284729)
Supplement: S3 Table — MIS: completed minimally invasive colectomy; CtO: minimally invasive colectomy converted to open; Open: planned open colectomy. Risk-adjusted estimates are reported as adjusted odds ratio (AOR) or ß-coefficients with 95% confidence intervals (CI) for binary and continuous variables, respectively. (DOCX) [file pone.0284729.s005.docx]

**Supplementary Table S3. Sensitivity Analysis: Risk-adjusted outcomes for patients with diverticulitis undergoing colectomy at Low Volume Hospitals stratified by operative approach.** *MIS: completed minimally invasive colectomy; CtO: minimally invasive colectomy converted to open; Open: planned open colectomy. Risk-adjusted estimates are reported as adjusted odds ratio (AOR) or ß-coefficients* with 95% confidence intervals (CI) *for binary and continuous variables, respectively.*

|  | **MIS** | **CtO** | **^a^P value** | **Open** | **CtO** | **^b^P value** |
| --- | --- | --- | --- | --- | --- | --- |
| Clinical Outcomes, AOR [95% CI] |  |  |  |  |  |  |
| In-Hospital Mortality | Ref | 0.5 [0.1, 2.5] | 0.4 | Ref | 0.4 [0.1, 1.3] | 0.1 |
| Cardiac Complications | Ref | 1.2 [0.5, 2.9] | 0.6 | Ref | 1.0 [0.5, 2.1] | 0.9 |
| Acute VTE | Ref | 1.1 [0.4, 2.5] | 0.9 | Ref | 0.7 [0.4, 1.4] | 0.3 |
| Respiratory Complications | Ref | 1.3 [0.9, 1.8] | 0.2 | Ref | 0.7 [0.5, 0.9] | 0.02 |
| Gastrointestinal Complications | Ref | 3.3 [1.8, 6.4] | <0.001 | Ref | 1.3 [0.8, 2.2] | 0.4 |
| Infectious Complications | Ref | 1.7 [1.3, 2.3] | <0.001 | Ref | 1.0 [0.7, 1.3] | 0.9 |
| Ileostomy Formation | Ref | 3.7 [2.9, 4.5] | <0.001 | Ref | 2.0 [1.6, 2.4] | <0.001 |
| Colostomy Formation | Ref | 4.0 [2.9, 5.5] | <0.001 | Ref | 1.0 [0.7, 1.3] | 0.9 |
| Resource Utilization, AOR/ ß-Coef [95%CI] |  |  |  |  |  |  |
| Length of Stay (days) | Ref | 1.8 [1.5, 2.0] | <0.001 | Ref | 0.2 [0.0, 0.5] | 0.056 |
| Hospitalization Costs ($1,000s) | Ref | 4.1 [3.1, 4.9] | <0.001 | Ref | 3.6 [2.7, 4.6] | <0.001 |
| Non-home Discharge | Ref | 1.9 [1.4, 2.5] | <0.001 | Ref | 0.8 [0.6, 1.0] | 0.09 |
| 30-day, Unplanned Readmissions | Ref | 1.3 [1.1, 1.6] | <0.001 | Ref | 1.2 [0.9, 1.4] | 1.2 |

^a^CtO vs. MIS

^b^CtO vs. Open
